# Interactions between a dsDNA Oligonucleotide and Imidazolium Chloride Ionic Liquids: Effect of Alkyl Chain Length, Part I

**DOI:** 10.3390/molecules27010116

**Published:** 2021-12-25

**Authors:** Fatemeh Fadaei, Michelle Seifert, Joshua R. Raymond, David Řeha, Natalia Kulik, Babak Minofar, Mark P. Heitz

**Affiliations:** 1 Laboratory of Structural Biology and Bioinformatics, Institute of Microbiology of the Czech Academy of Sciences, Zámek 136, 37333 Nove Hrady, Czech Republic; fadaei@nh.cas.cz (F.F.); reha@nh.cas.cz (D.Ř.); kulik@nh.cas.cz (N.K.); 2 Faculty of Science, University of South Bohemia in České Budějovice, Branišovská 1645/31A, 37005 Ceske Budejovice, Czech Republic; 3 Department of Chemistry and Biochemistry, SUNY Brockport, Brockport, NY 14420, USA; michelle.seifert@t-online.de (M.S.); jraym1@brockport.edu (J.R.R.)

**Keywords:** DAPI, DNA duplex, DNA oligonucleotide, imidazolium ionic liquids, fluorescence, fluorescence lifetime, molecular dynamics, radial distribution function

## Abstract

Ionic liquids (ILs) have become nearly ubiquitous solvents and their interactions with biomolecules has been a focus of study. Here, we used the fluorescence emission of DAPI, a groove binding fluorophore, coupled with molecular dynamics (MD) simulations to report on interactions between imidazolium chloride ([Im_n,1_]^+^) ionic liquids and a synthetic DNA oligonucleotide composed entirely of T/A bases (7(TA)) to elucidate the effects ILs on a model DNA duplex. Spectral shifts on the order of 500–1000 cm^−1^, spectral broadening (~1000 cm^−1^), and excitation and emission intensity ratio changes combine to give evidence of an increased DAPI environment heterogeneity on added IL. Fluorescence lifetimes for DAPI/IL solutions yielded two time constants 0.15 ns (~80% to 60% contribution) and 2.36–2.71 ns for IL up to 250 mM. With DNA, three time constants were required that varied with added IL (0.33–0.15 ns (1–58% contribution), ~1.7–1.0 ns (~5% contribution), and 3.8–3.6 ns (94–39% contribution)). MD radial distribution functions revealed that π-π stacking interactions between the imidazolium ring were dominant at lower IL concentration and that electrostatic and hydrophobic interactions become more prominent as IL concentration increased. Alkyl chain alignment with DNA and IL-IL interactions also varied with IL. Collectively, our data showed that, at low IL concentration, IL was primarily bound to the DNA minor groove and with increased IL concentration the phosphate regions and major groove binding sites were also important contributors to the complete set of IL-DNA duplex interactions.

## 1. Introduction

Deoxyribonucleic acid (DNA), the most important of biomolecules, plays pivotal biological roles in the storing and carrying of genetic information for the growth, functioning, and reproduction of all known organisms. Due to its tiny size, geometric properties, and atom recognition capabilities, DNA has been recognized as an interesting candidate for the development of new nanomaterial and advanced molecular devices [1,2]. For example, DNA probes, including synthetic oligonucleotides and amplicons, are used in a variety of DNA microarray and DNA chip devices for gene expression, point mutations, and pharmacogenomic and diagnostic applications [3]. Hence, due to the development of DNA molecular probes, the studies on the interaction between synthetic oligonucleotides and other molecules are very important. Recently, multiple works have investigated the interaction of novel molecules with DNA. For instance, Rezki’s group designed and synthesized the imidazolium ionic liquid halides carrying different fluorinated phenylacetamide side chains [4]. In other work, the same group studied pyridinium ionic liquids for their DNA binding properties [5]. In these examples, they investigated the interaction the synthesized ILs with DNA to determine the anticancer mechanism. According to their results, there was a reasonably good binding affinity between the DNA and the designed ionic liquids. In addition, Revathi et al. [6] synthesized a new series of bio active Cu(II) and Zn(II) complexes. They performed both experimental and theoretical study to investigate the possible interaction of them with DNA. They revealed that the mixed ligand complexes interact with CT DNA by intercalation mode. Besides, Alraqa and coworkers [7] designed and synthesized the hybridization of benzotriazole and 1,2,3-triazoles with several functionalities, including amide, ketone, ester, and/or carboxylic acid. According to their results, the compounds have good interaction with the minor grooves of DNA through hydrogen bonding and hydrophobic interactions. They also suggest the reported molecules may be future anticancer agents.

Ionic liquids (ILs) are made of organic cations and usually an inorganic anion, the combination of which results in a water-free salt with a melting point below 100 °C, as first defined by Walden in 1914 [8]. In consideration of using ILs as solvents, they offer attractive physicochemical properties, including low vapor pressure, high thermal stability, nonflammability, and good conductivity. As such, ILs have become viable alternative solvents or co-solvent to conventional organic solvents in different fields of life sciences. In addition, it is interesting to note that a wide variety of cations and anions can be used to form ILs and that each one has unique properties that can meet specific requirements for use in biochemistry, biotechnology, medicine, pharmacology, and bio-nanotechnology, etc., applications [9,10,11,12,13,14,15,16]. The applications and relevant functions of ILs in biomolecular systems are based on the nature of the specific interaction that occur between ILs and biomacromolecules. In recent years, researchers have started investigating interactions between ILs and biomolecules. For example, He et al. [17] used various experimental methods and molecular dynamics (MD) simulation to investigate the interactions between the imidazolium-based IL surfactant 1-dodecyl-3-methylimidazolium bromide [Im_12,1_]Br and DNA in dilute brine. According to their results, there are electrostatic attractions between DNA phosphate groups and [Im_12,1_]Br headgroups and hydrophobic interactions among the alkyl chains. Jumbri et al. [18] used both computational and experimental evidence to analyze the characteristics and the influence of ILs on the structural properties of DNA. The main criterion for DNA stabilization was the hydration shells around the DNA phosphate group where DNA maintains its duplex conformation in ILs. Wang and Cui [19] worked on the interaction of 1-butyl-3-methylimidazolium methanesulfonate ([Im_4,1_][MS]) and 1-hexyl-3-methylimidazolium methanesulfonate ([Im_6,1_][MS]) with calf thymus deoxyribonucleic acid (ctDNA) by fluorescence and ultraviolet–visible (UV) absorption spectra. According to their results, the binding force increased with increasing IL alkyl chain length. They also suggested that both electrostatic and hydrophobic interactions were present between the ctDNA with [Im_n,1_][MS], and hydrophobic interactions played a key role in the interaction process of ctDNA with IL.

**Scheme 1:**
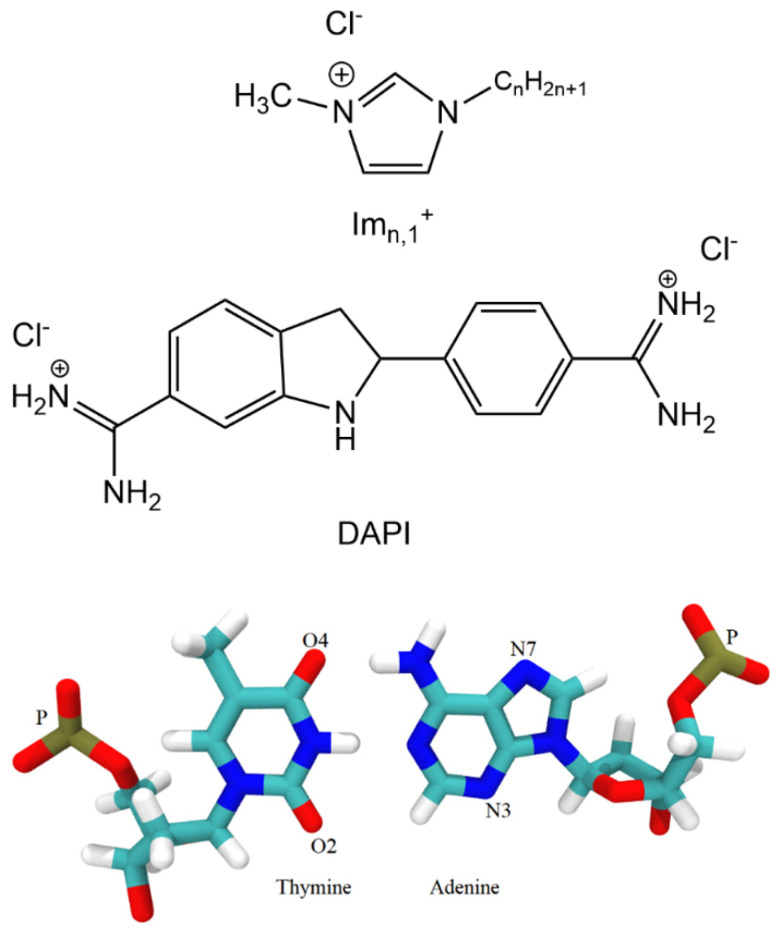
Structures of the 1- hexadecyl-3-methylimidazolium chloride ([Im_16,1_]Cl), 4, 6-Diamidin-2-phenylindol (DAPI), thymine, and adenine.

Since ILs play a major role in the search for potential candidates as replacements for organic solvents, which can have a deleterious impact on the environment and health, the assessment of ILs as potential green solvent candidates requires that a wide range of applications be examined and as mentioned these contexts must include a thorough examination of IL interactions with varieties of biomolecules. In this regard, it has been shown that ILs have the capability to stabilize DNA for periods of time up to a year, which makes them very attractive as potential solvents for DNA storage [2,20]. Spectroscopy offers a convenient and simple means by which DNA/IL interactions may be studied. Recently, methylene blue analogs have been studied with respect to aggregation and binding to salmon sperm DNA [21]. It was determined that arylamine fragments play a crucial role in DNA interactions, and judicious choice leads to control over binding. Additional work from the same group reported the interactions of PMAM-calix-dendrimers on DNA binding with no apparent effect on DNA structure that resulted in stabilized DNA [22]. A popular fluorescent probe is 4, 6-Diamidin-2-phenylindol (DAPI, Scheme 1), which is an A/T-nucleic acid specific fluorophore that has been shown to intercalate within the minor groove of B-form DNA [23,24,25,26,27,28]. Kapuscinskis’ research showed that the maximum amount of dye bound to synthetic DNA is one molecule of DAPI per three base pairs [23]. When DAPI binds to DNA, its fluorescence intensity increases and the absorbance shows a significant spectral red shift [29]. Thus, changes in the DAPI microenvironment can be readily detected by measuring spectral changes upon varying parameters that affect the DAPI–DNA interactions. In addition, time-resolved fluorescence has been used to examine DAPI interactions with solvents, polydeoxynucleotides, and linear and closed DNA [30,31,32]. Gratton and co-workers have reported on the lifetime response of DAPI in ctDNA, and several polydeoxynucleotides that included all A/T sequences polyd(AT), and poly(dA)poly(dT) [30,31,32]. They found that the decay kinetics in aqueous solution and alcohol solvents were best described by two time constants that were on the order of 0.2 ns and 2.5 ns, but, when in an A/T environment, the time constants lengthened to ~0.4 ns and ~3.8 ns. The fluorescence indicated a lifetime heterogeneity that had its origin from a distribution of ground state molecular conformers. Another conclusion drawn from this work is that the DAPI non-exponentiality is reflective of the DNA binding site heterogeneity [30,31]. They further discussed the idea of preferential solvation of the upon DAPI binding to DNA, where the degree of indole ring solvation is responsible for the relative contributions of the two observed time constants [30].

In the context of IL research, it is worth noting here that there is still little attention that has been given to the study of interactions between DNA and ILs. To that end, we studied IL effects on a model DNA oligomer system wherein we have examined the effect of IL concentration. The DNA oligonucleotide chosen has the single strand sequence 5′-TATATATATATATA-3′, (7(TA)). We have used steady-state and time-resolved fluorescence coupled with MD simulation to elucidate the interactions that occur between the 7(TA) and 1-hexadecyl-3-methylimidazolium chloride [Im_16,1_]Cl. In part, our goal is to provide data that can be used to aid in the selection of ILs to be used as solvent systems in a biomolecular context. While we report here in detail on a [Im_16,1_]Cl + 7(TA) system, we have also included in supporting information some preliminary results from MD simulations that compare [Im_16,1_]^+^ to [Im_10,1_]^+^ and [Im_4,1_]^+^ (Figure S1). We are at present in the process of submitting a second communication to report the experimental results using these shorter chain cations with the goal of providing an assessment of hydrophobicity (via carbon chain lengths variation) on the binding characteristics of ILs to DNA duplex.

## 2. Results

### 2.1. Fluorescence Measurements

#### 2.1.1. DAPI Binding Assay

**Figure 1:**
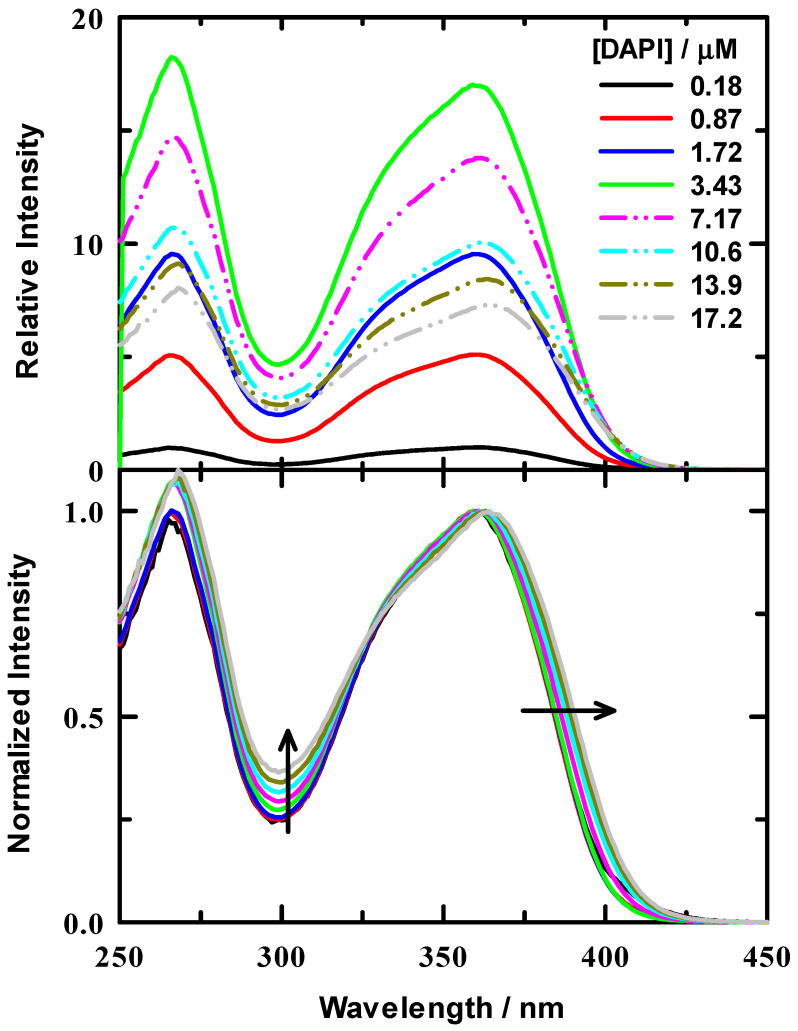
Excitation spectra for DAPI/DNA titration in 10 mM tris buffer at pH = 7.0. DNA concentration was 1.25 μM. Upper panel: Relative intensities of example spectra for varied ratios that are all scaled relative to DAPI/tris buffer. The DAPI/buffer was normalized to 1. Lower panel: normalized spectra for all ratios to help visualize the relative spectral changes. Arrows indicate the direction of spectral change with increasing ratio.

Initial measurements were performed to assess the binding of DAPI to a synthetic double stranded 7(TA) repeat sequence (14 base pairs per strand), B-DNA oligonucleotide to determine the optimal DAPI:DNA ratio be used in our experiments. Experiments with calf thymus DNA (ctDNA) suggested a binding ratio of between 0.04–0.06 dye:DNA base pair [20]. Other work suggested that the DAPI binding site size was 0.33 dye per base pair [23]. The latter work was based on 18 different polynucleotides rather than ctDNA. In this work, aliquots of a DAPI stock solution were titrated into a 1.25 μM DNA duplex/tris buffer solution. Excitation spectra from our titration are presented in Figure 1 for a representative series of DAPI concentrations. The upper panel illustrates the relative spectral intensities for the dye titration, where the spectra were normalized to the peak intensity of the 0.18 μM spectrum. It is well known that the DAPI fluorescence intensity increases when bound to DNA [23,26,27,29,30]. We observed that the fluorescence intensity of DAPI increases upon binding to our 7(TA) DNA duplex and reached a maximum and decreased when the oligonucleotide had accommodated the maximum number DAPI molecules. We observed a maximum increase of ~17-fold. Solid lines in these spectra are for data below the determined optimal binding concentration and dash-dot-dot lines are those above the threshold. In addition to these spectra, we also include normalized spectra in the lower panel of Figure 1 to highlight the spectral shift and shape change as DAPI was added. The normalized spectra show a red shift at the excitation maximum (~365 nm) and a systematic intensity increase, as indicated by the arrows. For completeness, we have also included a set of emission spectra at these same DAPI concentrations in Supporting Information Figure S2, which illustrate similar behavior to the excitation spectra, though the spectral red shift was more substantial.

**Figure 2:**
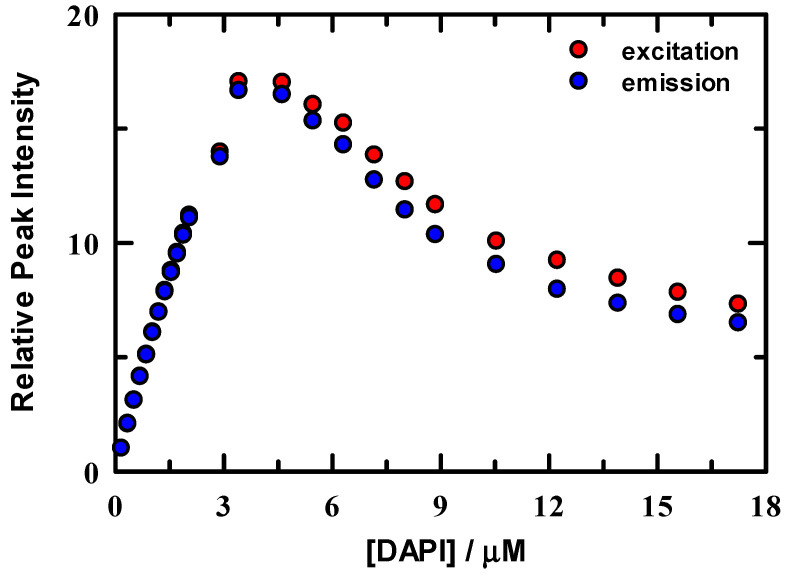
Normalized fluorescence intensities of DAPI in solution with 2.5 μM DNA/10 mM tris buffer at pH = 7.0. Data were normalized to the peak intensity for excitation (red) and emission (blue).

**Figure 3:**
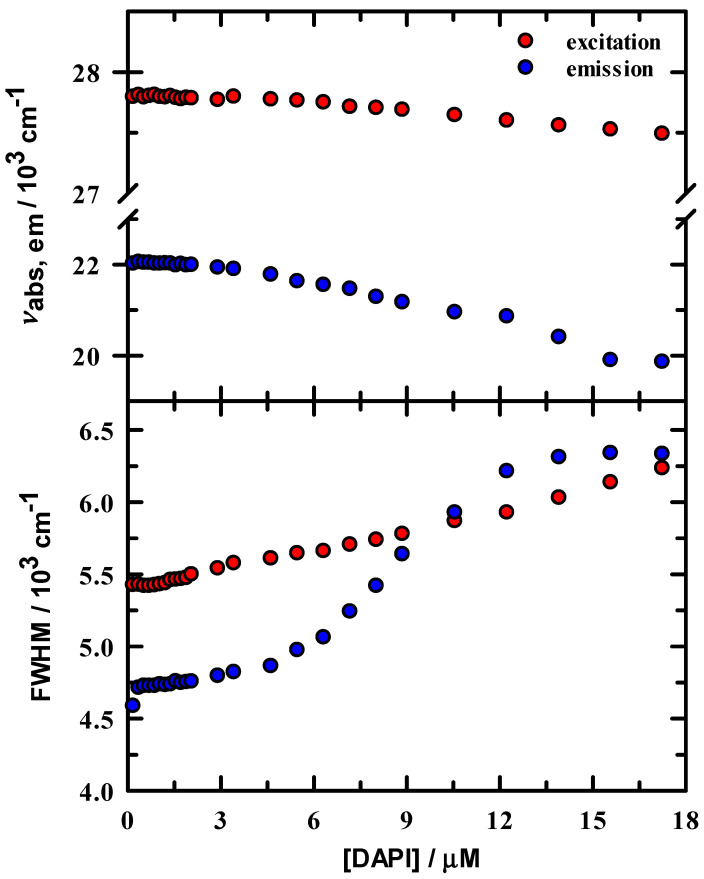
Steady-state spectral parameters for DAPI/DNA duplex in 10 mM tris buffer at pH = 7.0. Upper panel: excitation (red) and emission (blue) peak maxima. Lower panel: excitation and emission full width at half maximum values.

Excitation and emission peak intensities were normalized to the maximum value in the 0.18 μM spectrum and are shown in Figure 2. Intensity change is linear below ~3 μM DAPI and indicates that DAPI is continuing to bind to the DNA duplex up to this concentration. In either excitation or emission, the maximum intensity was located at ~3.5 μM DAPI, after which intensity systematically decreases. Figure 3 summarizes the energetics of the complete spectral results from the DAPI titration. The peak excitation (upper panel, red symbols) shifts only about 33 cm^−1^ up to a DAPI concentration of 2.1 μM, which is well under our experimental uncertainty of ~100 cm^−1^, whereas we observe a >300 cm^−1^ red shift for DAPI at 2.9–17.2 μM. In contrast, the emission (blue symbols) is more responsive to the DAPI changes, with peak shifts of 73 cm^−1^ and 2070 cm^−1^, respectively. In addition, included in Figure 3 are the excitation and emission spectra full width at half maximum values (FWHM, lower panel). The FWHM show nominal change up to ~3 μM DAPI, and the spectra broaden significantly over the remainder of the titration. Here, DAPI is experiencing much broader range of interactions that include at least DAPI–DNA and DAPI-solvent varieties. From these spectroscopic measures, we estimate that the DNA duplex reaches maximum binding at ~ 3.5 μM, most clearly seen in Figure 2. To facilitate comparison of our data with Kapuściński [23], we assumed that our binding concentration estimate could be a representative average over the 14-base pair DNA, but we also exclude the two end TA pairs as less probable binding locations because of extra degrees of motional freedom. To this end, we compute a crudely estimated binding ratio of 0.29 DAPI per base pair and find that this value is in reasonable agreement with Kapuściński’s ratio of 0.33 DAPI per DNA base pair. We also estimated the association constant from the fluorescence data using the method described by Boger and co-workers [33] and determined a value for K of 2.8 × 10^6^ M^−1^ in general agreement with DAPI literature reports that studied synthetic DNA oligomers [23,26,27,33].

#### 2.1.2. Steady-State Spectroscopy and [Im_16,1_]Cl Titration

**Figure 4:**
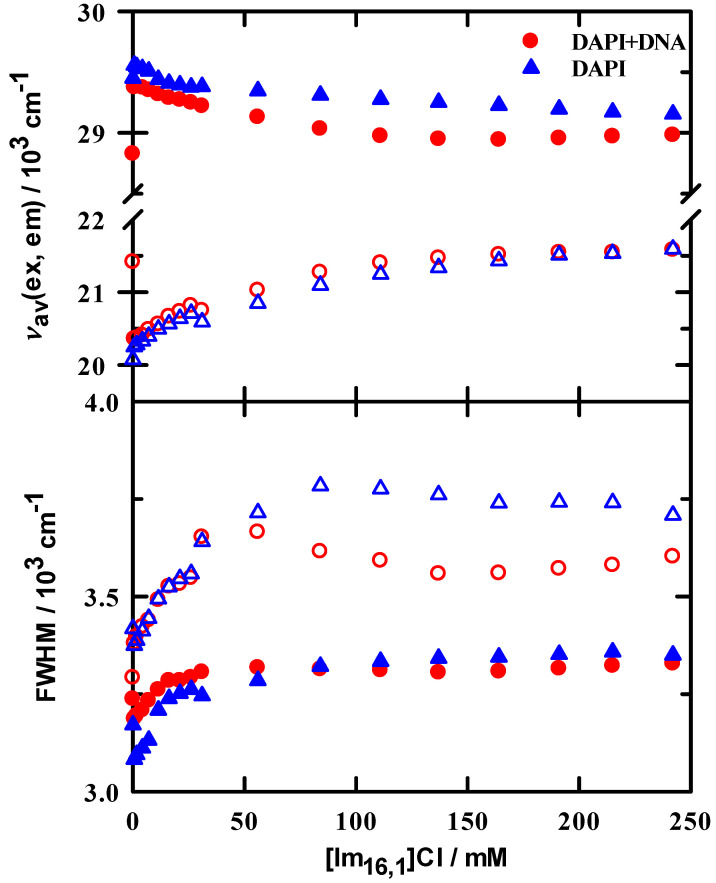
Steady-state excitation (filled symbols) and emission (open symbols) for DAPI + DNA duplex (circles) and DAPI (triangles) in 10 mM tris buffer at pH = 7.0 as a function of added IL. Upper panel: peak positions calculated from spectral first moments (intensity weighted average frequencies). Lower panel: full width at half maximum values of the intensity weighted spectra.

Excitation and emission spectral features upon addition of IL are similar to those observed in the DAPI + DNA duplex binding measurements. Spectral examples are shown in supporting information; see Figures S3 and S4. The effects of IL addition on steady-state spectroscopy are also summarized in Figure 4, where we show the first and second moments (intensity weighted average frequency and width, respectively) of the excitation and emission spectra. The spectral first moment is selected as our metric because it also captures the variability in spectra shape in addition to the simple peak position. Thus, the trends in these data are more representative of the average environment probed by DAPI as IL is added to solution. Data are shown for DAPI in the absence (▲,∆) and presence of DNA duplex (●,○). For excitation, we observe a small red shift in the first moment of approximately 300 cm^−1^, compared to the more substantial shift of 1000 cm^−1^ for the unweighted peak maximum. The moderation of the first moment shift is explained by the increased intensity of the 265 nm peak. In the presence of DNA duplex, we also observed red shifts of similar magnitude, 400 cm^−1^ in the first moment, and 950 cm^−1^ for the ‘360 nm’ peak maximum. What is of particular interest here is that the shift with DNA results from an initially lower energy in the absence of IL (●) and immediately blue shifts on the first IL addition, followed by a gradual red shift to a constant position. It appears from the excitation data that, while DAPI is bound to DNA duplex, it immediately senses the presence of IL and shifts its energy to nearly the value of DAPI + buffer + IL. A similar pattern is observed for the emission data, where a strong initial red shift in the DAPI + DNA duplex emission occurs with added IL, followed by a smoothly increasing blue shift. The widths in both excitation and emission for both DAPI and DAPI + DNA duplex consistently increased, indicating an increased solution heterogeneity in the presence of IL.

**Figure 5:**
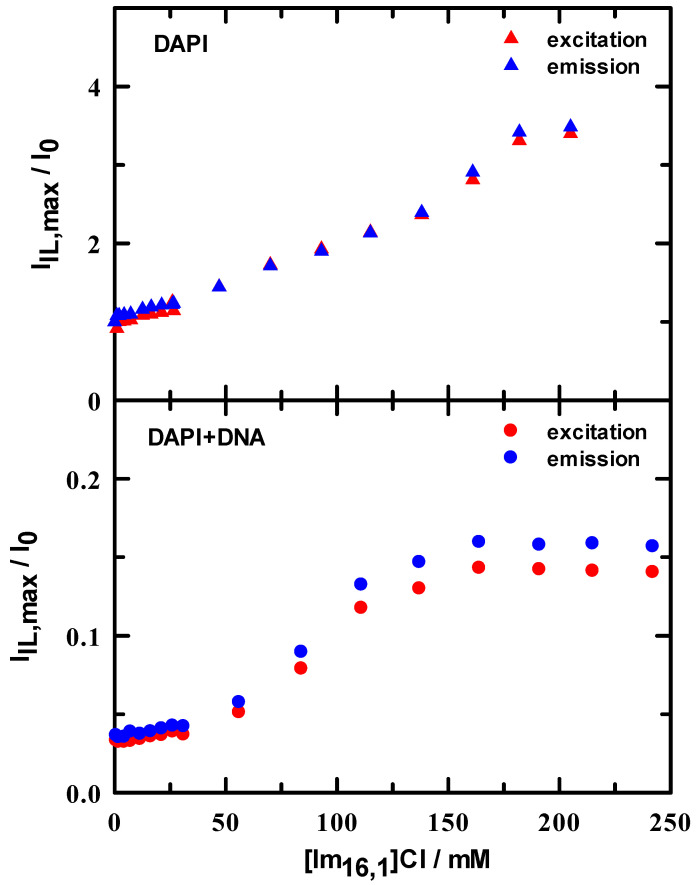
Spectral intensity ratios in 10 mM tris buffer at pH = 7.0 as a function of added IL determined by ratioing intensity in the presence of IL to that in the absence of IL. Upper panel: DAPI excitation (▲) and emission (▲) ratios. Lower panel: DAPI + DNA duplex excitation (●) and emission (●) ratios.

As a further metric for the behavior of spectral peak positions, we computed two sets of peak ratios as a simple means by which we tracked changes on IL addition. The excitation peak ratio (I_360_/I_265_) within a single spectrum at each IL concentration (see Figure S5) gives a sense of whether there is any band sensitivity to changes in IL concentration. Initial ratio values are 1.6 and 1.0 for DAPI in tris buffer and DAPI + DNA duplex in tris buffer, respectively. In brief, these ratios both steadily decrease over an IL range of 0–75 mM and then remain essentially constant to beyond 200 mM. Using this measure, other than a nearly uniform intensity difference over the entire IL concentration range, there appears to be nothing special about this ratio. The second ratio uses the spectral maximum at approximately 360 nm for excitation and 460 nm for emission, and computes values for I_[IL]_/I_[IL]=0_ as IL concentration is varied. These ratios are illustrated in Figure 5 and show rather different behaviors. In DAPI/buffer solutions, the minimum ratio is 1 and rises nearly linearly by more than a factor of 3, independent of excitation or emission. In stark contrast, the DAPI + DNA duplex ratios are all less than ~0.15 with a constant value up to ~50 mM, followed by a steep increase between 50–150 mM and then remain constant. There is a small difference between excitation and emission values, but the series clearly parallel one another.

#### 2.1.3. Time–Resolved Spectroscopy and [Im_16,1_]Cl Titration

**Figure 6:**
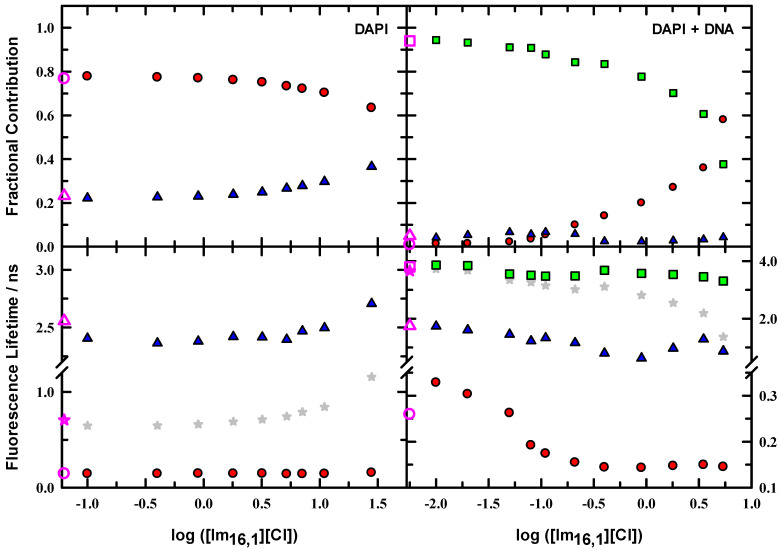
Lifetimes and fractional contributions for DAPI and DAPI + DNA duplex in 10 mM tris buffer at pH = 7.0 as a function of added IL. Left panels: DAPI time constants and associated fractions for a two component fit to the data (●,▲). Right panels: Data for DAPI + DNA duplex for a three component fit to the data (●,▲,■).

While the steady-state spectroscopy provides a general characterization of the DAPI fluorescence characteristics, time-resolved fluorescence reveals further details of the emissive properties and compiles the details of magic-angle intensity decay measurements for DAPI and DAPI + DNA duplex solutions in the presence of IL; Figure 6 illustrates the variations as IL is added to solution. These data are presented using a logarithmic scale to show the parameter variation more clearly, particularly at low IL concentration. Filled symbols show the data for time constants and associated fractional contributions to the intensity decay for solutions that include IL, and open pink symbols show the corresponding data in the absence of IL for comparison. Fractions were calculated from the raw data as

(1) $$f_{i}=\frac{a_{i}\tau_{i}}{\sum^{\;}a_{i}\tau_{i}}$$

where *a_i_* is the normalized un-weighted pre-exponential factors determined from the DAS-6 fitting parameters, and *τ_i_* is the *i*^th^ component lifetime. Star symbols represent the fractionally weighted lifetimes, which are computed as follows:

(2) $$\langle \tau \rangle =\frac{\sum^{\;}a_{i}\tau_{i}^{2}}{\sum^{\;}a_{i}\tau_{i}}$$

with parameters defined as in Equation (1). We see from the DAPI data (left panels) that the decays in all solutions are well described by two time constants with the larger contribution from the faster time constant (~140 ± 15 ps). The slower time constant varies between 2.36–2.71 ns. Neat buffer gives a reference point for the emission, and we see that the relative contributions are 77% and 23% for the faster and slower times, respectively. Upon addition of IL, the data shows an increase in the slower lifetime of ~13%, most evident beyond ~3 mM (~log (0.5)), while the faster lifetime remains essentially constant. The slower time contribution drops to 63%. Here, the trend in average lifetime tracks well with the slower time constant.

When DNA duplex is included in solution, three time constants are needed to characterize the intensity decay (Figure 6, right panels, note the lifetime scale change denoted on the right axis of the lower panel). In buffer solution, the faster (●) and middle (▲) time constants are similar to the DAPI/buffer values but show a modest change, where the fastest time constant increases 260 ps, and the second time decreases to ~1.7 ns. The slowest time constant (■) is 3.8 ns. DNA duplex in solution re-balances the relative contributions, and the two faster times contribute a combined 6% to the total decay. DNA-bound DAPI is dominated by the 3.8 ns lifetime. Since the DAPI/buffer solution requires two time constants, the inclusion of a third time constant with DNA duplex in solution suggests that the three time constants collectively describe two DAPI microenvironments. Moreover, the 94% contribution for the 3.8 ns indicates that DAPI is not 100% bound to DNA duplex, and some free DAPI is in solution. On addition of IL to 10 μM, only the fastest time constant changes and increases to 330 ps with the same relative contribution. By 400 μM IL, the fastest time constant has decreased to the level of DAPI/buffer at ~150 ps and remains at that value. The middle time constant generally decreased over the IL range measured, as did the slowest time constant. The fractional contribution changes are also important, and both the fastest and slowest values showed steady change with IL such that, in the 5 mM IL solution, 58% of the intensity decay was from the 145 ps component, and 38% was from the 3.31 ns. These changes signal that the IL is effectively manipulating the DAPI microenvironment. The average lifetime values also indicate these changes and show that the most precipitous decrease begins at about 400 μM.

### 2.2. MD Simulations

#### 2.2.1. The Effect of Concentration on Binding Characteristics of ILs–DNA Duplex

**Figure 7:**
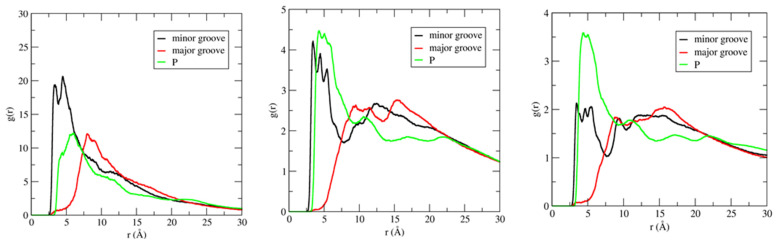
Center–of–mass (COM) radial distribution functions (RDF) for the [Im_16,1_]^+^ imidazolium ring around phosphate groups, minor, and major grooves of DNA duplex in different IL concentration solutions. RDFs calculations are performed using the N7 and O4 atoms for the electronegative sites in the major groove. Electronegative sites for the minor groove use N3 and O2 atoms. P atoms were used for the phosphate groups. IL concentrations are 20 mM (**left panel**), 250 mM (**middle panel**), and 500 mM (**right panel**).

IL cation radial distribution functions (RDFs) have been calculated in three different regions of DNA duplex, which include the phosphate backbone, major groove, and minor groove, to investigate the binding pattern(s) of ILs to DNA duplex. Figure 7 shows the distribution of the imidazolium ring center-of-mass (COM) around the phosphate backbone, major groove, and minor groove of DNA duplex, at different IL concentrations.

For the RDF calculations, we used the phosphate group P atom, electronegative sites N3 and O2 for the minor groove, and electronegative sites N7, O6, and O4 for the major groove (see Scheme 1 for atom labeling). RDFs provide evidence that demonstrates IL cations occupy space within the DNA backbone and groove regions. There is preferential accumulation of the imidazolium ring COM in the minor groove (black lines) at low IL concentration (20 mM, left panel) by a factor of two compared to the major groove (red lines) and P regions (green lines), implying the intrusion of IL cation into the DNA duplex groove. The large peaks for the minor groove−COM of the [Im_16,1_]^+^ imidazolium ring locates the COM at 3.4 Å. The position of the peak also gives the nature of the interactions, and the DNA duplex–IL interaction here is primarily a π-π stacking interaction. Simulated data for the other ILs yield similar results in dilute solution (see Figure S6). Furthermore, the shift to longer distance of the phosphate − IL and major groove − IL peaks from 3.4 Å is due to the unavailability of space filled by ILs cations. The same behavior has been repeated in dilute solution by computing the RDFs of IL cation alkyl chain in the three regions of the DNA duplex. By increasing the [Im_16,1_]^+^ concentration, the IL cations also established a significant interaction with the DNA phosphate groups (Figure 7, middle and right panels), the interaction of which is primarily electrostatic between the positively charged imidazolium ring and the negatively charged phosphate.

**Figure 8:**
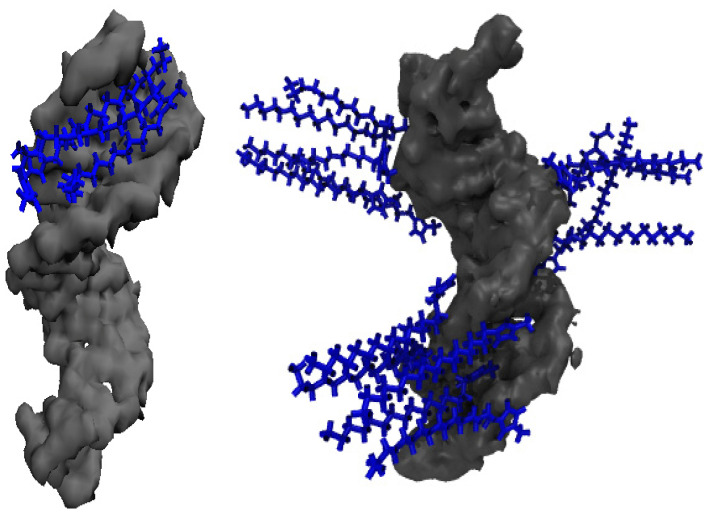
(**Left image**): The distribution of [Im_16,1_]^+^ cations (blue color) that shows the association with DNA duplex (gray) minor grooves in the aqueous solution. IL concentration is 20 mM. (**Right image**): The distribution of [Im_16,1_]^+^ cations for the association with the DNA duplex surface in the aqueous solution at 500 mM.

Figure 8 provides a view of the cation alignment in the DNA duplex minor groove in the 20 mM IL solution (left panel) and a representative snapshot of these cations that align perpendicular to the DNA duplex surface in 500 mM concentrated IL solution (right panel).

These findings are corroborated by a report from Ding et al. [34], who investigated the binding characteristics and molecular interaction mechanism between [Im_4,1_]Cl and DNA in aqueous solution medium used a wide variety of measurement techniques that included conductivity measurements, fluorescence spectroscopy, dynamic light scattering (DLS), cryogenic transmission electron microscopy (cryo–TEM), circular dichroism spectroscopy, ^31^P nuclear magnetic resonance (NMR) spectroscopy, Fourier transform infrared spectroscopy, isothermal titration calorimetry (ITC), and quantum chemical calculations. Based on their reported quantum chemical calculations, at a low IL concentration, the cationic headgroups of [Im_4,1_]Cl localized within several angstroms of the DNA phosphates, whereas the hydrophobic chains arranged parallel to the DNA surface. When the IL concentration is above 0.06 M, the cationic headgroups are near DNA phosphates, and the hydrocarbon chains are perpendicularly attached to the DNA surface.

**Figure 9:**
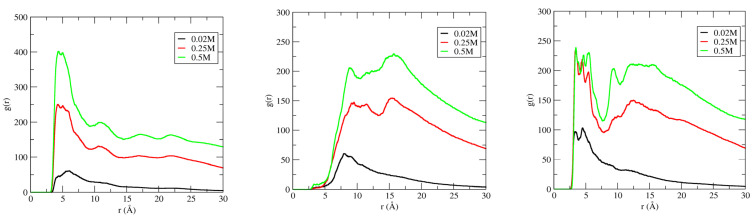
Concentration dependence of the [Im_16,1_]^+^ imidazolium ring center-of-mass distribution functions around the phosphate backbone (**left panel**), major groove (**middle panel**), and minor groove (**right panel**) of DNA duplex in aqueous solutions. IL concentrations are 20 mM (black line), 250 mM (red line), and 500 mM (green line) in each panel.

Owing to the perpendicular orientation of [Im_16,1_]^+^ to the DNA duplex groove bases, the IL alkyl chains on the opposite face of the imidazolium are exposed to the bulk solution and parallel alkyl group alignment of the cations to each other implies the existence of alkyl–alkyl chain interactions. The imidazolium ring COM RDFs around the DNA duplex were modified by multiplying the RDF at each concentration by the average number density of particles in the system, with the goal of determining the effect of concentration on the binding characteristics of IL–DNA duplex. Figure 9 shows the concentration dependent results for the imidazolium ring COM distribution around the DNA duplex phosphate backbone (left panel), major groove (middle panel), and minor groove (right panel) for 20 mM, 250 mM, and 500 mM solutions.

**Figure 10:**
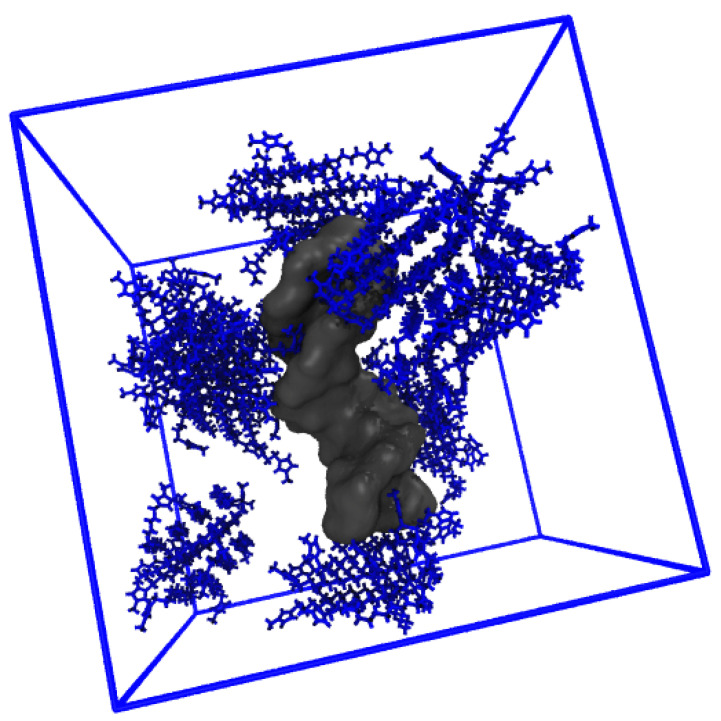
Aggregation of [Im_16,1_]Cl at 500 mM.

The distribution of IL cations around the various DNA duplex regions increases significantly with increasing IL concentration in all cases. In the P region, there is an 8-fold increase in *g*(*r*) from 20 to 500 mM, and a ~4.5-fold increase and ~2.5-fold increase for the major and minor groove populations, respectively. Further, for P, the IL remains localized at ~5 Å as concentration is increased, but, for the DNA duplex groove regions, the dominant contribution shifts consistently from ~5 Å to 15 Å, where the *g*(*r*) values are approximately equal. In addition, we note further that IL aggregation also occurs at high IL concentration, and this is pictured in Figure 10.

#### 2.2.2. The Effect of Alkyl Chain Length on the Binding Characteristics of ILs–DNA Duplex

**Figure 11:**
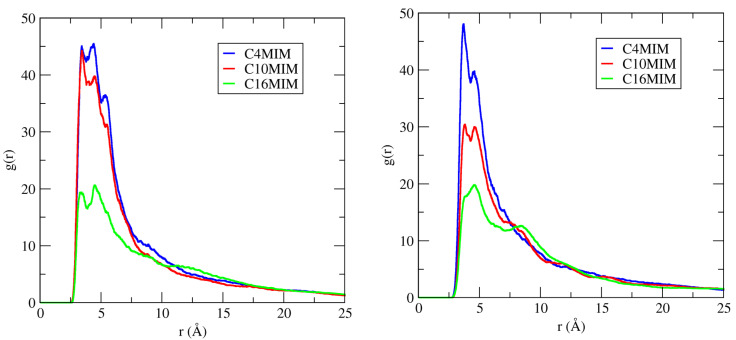
RDFs of the center-of-mass around minor grooves of DNA duplex in 20 mM IL solutions. The electronegative sites N3 and O2 atoms for the minor groove were used for these RDF calculations. (**Left panel**): Data for the imidazolium ring–groove interactions. (**Right panel**): Data for tail–groove interactions.

The COM RDF of the cation ring and cation alkyl chain around the DNA duplex surface were calculated to characterize more completely the binding characteristics of ILs to DNA duplex. According to Figure 7, in dilute solution (IL = 20 mM), the COM of the imidazolium ring in hydrated ILs interacts most frequently with the DNA duplex minor groove. Figure 11, left panel, shows the COM RDFs of the imidazolium ring around DNA duplex minor grooves in 20 mM IL solutions.

Here, we see that, by increasing the length of cation alkyl chains from C4 to C16, the COM distribution of the imidazolium ring around minor grooves of DNA decreases (left panel). We also illustrate the cation alkyl chain RDFs around the DNA minor grooves in 20 mM IL (Figure 11, right panel), and they, too, also decrease with chain length. According to these data, there is less interaction probability between the longer alkyl chain IL and the DNA minor groove.

## 3. Discussion

Two different binding modes have been reported from force spectroscopy measurements of DAPI binding to DNA duplex under varying ionic strength [35]. The authors report that DAPI minor groove binding occurs first and is the dominant mode until DNA duplex saturation is achieved, with a reported binding constant about 10^7^ M^−1^. The second binding mode is reported as intercalation, which is only observed after DNA saturation and with a decreased binding constant of 10^5^ M^−1^. Our spectroscopic results are consistent with these modes of association, as evidenced by the contrast in IL solution intensity ratios between DAPI and DAPI + DNA duplex (see Figure 5). For DAPI/IL solutions, there is no specific binding mechanism, and the ratio increases linearly with IL concentration, whereas, when DNA is present, the intensity ratio is suggestive of an IL concentration induction at low IL and presents a sigmoid shape as IL concentration increases. At least two possibilities explain this observation, where, in the first case, the IL can displace the bound DAPI but only after a threshold concentration reaches, seemingly, ~30–40 mM IL. At high IL concentration, the IL–IL associations offers an additional milieu (see MD results) with which DAPI is likely interacting, in addition to a combined DNA duplex-IL environment. For example, [Im_16,1_]Cl has been shown to undergo aggregation in aqueous solution. El Seoud and co-workers determined the critical micelle concentration (cmc) for [Im_16,1_]Cl from conductivity, surface tension, and fluorescence measurements and reported a cmc value of ~0.9 mM [36,37]. Therefore, [Im_16,1_]Cl in our solutions has clearly aggregated. However, the consistent response of DAPI over the entire titration range reported here indicated that, while IL aggregation occurs, it does not appear to affect the DAPI spectroscopy in any obvious manner. In addition, since DAPI is a dicationic dye, it must localize near to the IL ion region and, as such, resides in the aqueous-IL interfacial region. More importantly, when in the presence of DNA, DAPI is strongly associated with the duplex minor groove, and IL aggregation that occurs in the bulk does not appear to interfere with this spectroscopic signature. In fact, except for the difference in absolute value of the intensity ratios (see Figure 5), we calculate the same values for slope (i.e., same degree of linearity) when normalizing the intensity ratios in the lower IL concentration range of 0–30 mM for DAPI, in either the absence or presence of DNA. Thus, it is the DAPI–DNA interaction that is the primary driver of the observed intensity ratio difference and the presence of DNA effectively mitigates the impact of IL on the DAPI spectroscopy. The intensity ratio for DAPI/IL is between 1 and 4, whereas, in the presence of DNA, duplex is not more than 0.15, even at 200 mM IL. The intensity ratio increases and signals that DNA duplex bound DAPI does sense IL but, apparently, does not see a neat IL environment.

In consideration of the lifetime data, the fastest (●, Figure 6) time constant change on first addition of IL (=10 μM) gives evidence that the IL interacts with DAPI. However, more importantly, since the time constant value increased, we interpret this as the IL interacting with DNA duplex-bound DAPI because the fastest time constant for the free DAPI-IL interaction was 145 ps and remained constant independent of IL. With successive additions of IL to the DNA duplex solution, we noted a steady decrease in the fastest time constant (●) with a concomitant increase in its fractional contribution, with a simultaneous reduction in the contribution of the slowest time constant (■), which suggests a free DAPI environment is increasingly observed with added IL. It may be that IL has saturated the DNA duplex bound DAPI population and quenched the fluorescence, thereby shortening the DAPI lifetime, or that IL is displacing DAPI from the DNA duplex minor groove binding site, and the proportion of free DAPI fluorescence has increased. In either case, the fractional contribution (●) changes buttress the free DAPI perspective since the value increases dramatically from 1% in DAPI–DNA/buffer to 58% in 5 mM IL solution.

The data obtained from steady-state spectroscopy for [Im_16,1_]Cl titration indicated a small red shift in the first moment of approximately 300 cm^−1^ and also revealed that DAPI bound to the DNA duplex immediately sensed the presence of IL. The energy shifted to nearly the value of DAPI + IL+ buffer. This observation is consistent with the interactions revealed from MD simulations and corroborates the MD results. Of course, the MD data cannot be quantitatively compared with the spectroscopic data, but the RDF analysis revealed that such interactions are indeed present in the DNA + IL solutions. From MD simulations, we noted that, in the most dilute solution (20 mM), the hydrophobic alkyl chain is arranged parallel to the DNA duplex surface, where the imidazolium ring interacts via π-π stacking interaction with grooves. Thus, the interactions between DAPI and IL are projected to be the greatest. In the concentrated solution (500 mM), the cationic headgroups are near the DNA duplex phosphates, and the hydrocarbon chains are perpendicularly attached to the DNA duplex surface, especially in hydrated [Im_16,1_]^+^. We have concluded that the hydrophobic interaction between the IL hydrocarbon chain and the bases of DNA duplex plays a dominant role at lower IL concentrations, and the electrostatic interaction is essential in the IL binding to DNA duplex at higher IL concentrations. In the most dilute solution (20 mM), the hydrophobic alkyl chain is arranged parallel to the DNA duplex surface where the imidazolium ring interacts via π-π stacking interaction with grooves. The RDF data suggest that shorter chain cations readily populate the DNA duplex minor groove compared to longer chain analogs.

## 4. Materials and Methods

Chemicals were purchased and prepared as follows. The DAPI dye was purchased from Invitrogen ThermoFisher Scientific, Life Technologies Corporation, Carlsbad, CA, USA, and prepared at a stock concentration of 1 mM. The DNA oligonucleotides were from Integrated DNA Technologies, Inc. (IDT), Coralville, IA, USA. A 0.5 mM stock solution of dsDNA (1 mM single strand) was prepared and used in dilution to make all required oligonucleotides solutions. Tris-HCl buffer (10 mM, pH 7) was used as a solvent. ILs were purchased from Ionic Liquids Technologies (IoLiTec), Heilbronn, Germany at >98%, stored in a nitrogen glove box and used as received.

The UV-visible (UV-vis) absorption spectrum was measured using a Perkin-Elmer double beam Lambda 800 UV-vis spectrophotometer, Waltham, MA, USA. Absorption measurements were made by scanning between 220 to 450 nm, with a slit width of 2.0 nm. Steady-state excitation and emission spectra were measured using a HORIBA Fluorolog 3 fluorimeter, Horiba Scientific, Piscataway, NJ, USA. The fluorimeter is equipped with a single grating excitation monochromator and a double grating emission monochromator for enhanced stray light rejection. All spectra were subtracted and corrected for instrument responses. Excitation spectra were measured over a wavelength range of 250–450 nm with emission detected at 470 nm, whereas emission spectra were measured by scanning over a wavelength range of 350–650 nm with excitation at 330 nm. For both spectral acquisitions, excitation and emission slits were fixed at a 2 nm band pass. When the signal was too high for the detector to respond linearly (>2 × 10^6^ cps), the signal was reduced using a spectrally flat neutral density filter that applied a 2.93 factor reduction in intensity. Spectra were corrected when needed and the re-scaled data were used in the data set. We have described our time-correlated single photon counting (TCSPC) instrument previously [38,39]. Briefly, photons from a 405 nm NanoLED (405-L) high output diode laser (Horiba Scientific, Piscataway, NJ, USA) were passed through a polarizer prior to entering the sample. Emission photons were passed through an automated Glan-Thompson polarizer set at “magic” angle (54.7°) for lifetime measurements and spectrally resolved with the double grating monochromator, as well as detected with an air-cooled IBH TBX 850 detector. The instrument response function was measured using a scattering solution and was on the order of 170 ps. Time calibration of the counting electronics was 7.16 ps per channel. All decay data was measured at the peak of the steady-state emission with emission slits between 4–6 nm. Intensity decays were fit using a sum of exponentials models with an iterative reconvolution algorithm that was part of the IBH DAS6 decay analysis software (Horiba Scientific, Piscataway, NJ, USA). Following the deconvolution fitting process, the estimated effective time resolution was ~50 ps, which we have also confirmed from replicate measurements. Reduced chi-squared values (*χ*_r_^2^) for a fit was judged to be acceptable if *χ*_r_^2^ < 1.2.

For DAPI-DNA duplex binding experiments, we used we used a 1.25 μM dsDNA sample with a constant volume addition of DAPI titrated into solution that resulted in a concentration that ranged from 0.175 to 17.2 μM DAPI in tris-HCl buffer. A solution of 1.25 μM dsDNA in tris-HCl buffer was used as the blank. For IL experiments, three solutions were prepared and run in parallel. The first set of solutions was used to measure the response of DAPI to the presence of ILs. A second set of solutions were made that included 1.25 μM of the DNA oligonucleotide. Finally, tris-HCl buffer containing the corresponding amount of IL was used as a blank. The IL concentrations added to each sample ranged from 0–250 mM. All optical measurements were made in 1 cm quartz cuvettes (23-Q-10, Starna Inc, Atascadero, CA, USA).

MD simulations were performed using the General Amber Force Field (GAFF) [40] and AMBER99SB-ILDN protein, nucleic AMBER94 [41] were used for the imidazolium-based ILs and DNA duplex, respectively. To build the initial structure of the systems, the DNA duplex molecule was placed in the center of the cubic box with the size of 7.1 × 7.1 × 7.1 nm. A total number of 5, 56, and 112 pair of cations and anions were added randomly by Packmol package in the simulation boxes [42,43] to reach the desired IL concentrations from the experiment that were 0.02 M, 0.25 M, and 0.5 M. Then, the simulation boxes were solvated by water molecules, using the TIP3P model of water [44]. As certain undesirable interactions might arise in the systems due to randomly added molecules to the simulation boxes, the steepest descent minimization approach was utilized to eliminate all unfavorable interactions. After that, all systems were equilibrated by performing 100 ps NVT (Canonical ensemble) restrained simulations followed by 100 ps NPT (isothermal–isobaric ensemble). Equilibration proceeded with the production runs where the linear constraint solver (LINCS) algorithm [45] was employed for all bonds involving hydrogen atoms and short-range non-bonded interactions were cut off by 1.2 nm. Long-range electrostatic interactions were treated by the particle mesh Ewald method [46] procedure. To produce initial velocities, Maxwell–Boltzmann distribution was used for all simulations. V-rescale coupling algorithm was used [47] with the coupling constant of 0.1 ps to ensure constant temperature and pressure during the simulations. MD production runs were performed for 200 ns at 300 K, where 2 fs time step was used. Data for further analysis were stored in every 5 ps for all simulations. The GROMACS 2018 program package was used for performing MD simulations [48,49,50,51,52]. In addition, Visual Molecular Dynamics (VMD) was applied for visualizations and preparation of snapshots [53]. After the simulation, the radial distribution function (RDF) was used to describe the distribution of solvent molecules around the specific molecule or atoms of DNA or ILs.

We note here that using only picoseconds of equilibration time may be insufficient for the complex solutions studied here; therefore, we analyzed the data using the last 150 ns of the 200 ns simulation, which means we used the first 50 ns as an equilibration phase. The equilibration process described above was also checked by running an initial 10 ns NPT equilibration, followed by a 10 ns NVT equilibration, before starting the production run. A comparison between using the standard GROMACS procedure and using this longer equilibration process and running an initial 10 ns NPT equilibration, followed by a 10 ns NVT, both led to equilibrated systems which could be used for further analysis. Moreover, using either equilibration procedure did not change the final conclusions reached from the MD calculations. With regard to simulation equilibration, achievement of equilibrium/structural stability was checked by evaluating the root-mean-square deviation (RMSD), and Figure S7 shows examples of RMSD values for aqueous and IL solutions.

Additionally, Figure S8 shows unsmoothed RDFs for IL tail–DNA groove interaction in 20 mM IL to provide a sense of the actual fluctuations in the RDF data. Otherwise, all other RDF plots here are presented as smoothed functions.

## 5. Conclusions

Millions of biological samples, including cells, and DNA/RNA, are stored every year for diagnostics, research, and forensic science. Solvents play a critically important role in various ways from stabilizing and preserving the conformation of DNA to storage and handling of DNA, with application to different fields, including biology, medical diagnostics, and biotechnology. DNA samples in aqueous solution cannot preserve their structures for long periods of time and degradation of the DNA structure after prolonged storage is observed. In addition, there is the risk of hydrolytic and oxidative damage for DNA in water solutions. Moreover, using pure water as a solvent for DNA creates some difficulties in nanotechnology due to water vaporization for samples in small volumes and when under open-air conditions. Hence, to increase the use of DNA in industry, searching and finding an appropriate medium to increase the stability of DNA and overcome the limitations of aqueous buffers is necessary. Aqueous imidazolium based ionic liquids are suggested as medium to enhance the structural stability of DNA. However, the interaction mechanism between this IL class and DNA has been not completely assessed.

We have reported on the interactions between a self-complimentary 7(TA) DNA oligonucleotide and imidazolium chloride ionic liquids using steady-state and time-resolved fluorescence spectroscopy and complimented the experimental data with molecular dynamics simulations. We first determined the binding ratio between the fluorescent dye, DAPI, and the DNA duplex and found that there were approximately 3 DAPIs per DNA (here, DAPI ~3 μM), in good agreement with literature reports. Steady-state spectroscopy showed that, beyond 3 μM DAPI, the excitation and emission spectra blue shifted and broadened significantly, indicating that DAPI was both bound to DNA but also interacted with bulk solvent. In the presence of DNA + IL, the excitation and emission spectra showed an immediate shift, which then moderated back toward the initial value observed in the absence of IL. Spectra remained broadened over the range of IL concentration, confirming that DAPI was residing in multiple environments. Computed intensity ratios (I_[IL]_/I_[IL] = 0_) as a function of IL showed clearly different values for DAPI + IL solutions (~1–4) and DAPI + DNA + IL (<0.15), which differentiated DAPI interactions with bulk IL from the DNA-IL interactions. While the ratio values suggest that IL likely displaces some DAPI from its minor groove binding location (e.g., increased ratio), the DAPI appears to largely remain in the DNA groove.

Fluorescence lifetimes showed that, for DAPI in IL solutions, two time constants were required, with about 78% of the intensity decay associated with a 150 ps lifetime, and the contribution moderated to about 60% in 30 mM IL. The longer lifetime varied from 2.4 ns to 2.7 ns over the same range. The average lifetime varied from 0.7 ns to 1.1 ns. For DNA–based solutions, three time constants were needed, two of which were similar to DAPI/IL, with the addition of third time of ~3.8 ns. The contribution of the fastest time decreased from 330 ps to 150 ps, with a contribution that rose from an initial value of 1% to 58% in 5 mM IL. Moreover, the longest lifetime, associated with the DAPI–DNA duplex interaction, remained similar in time but decreased in contribution from 94% to 39%. We take this to indicate that DAPI senses an increasingly bulk–like IL environment while still associating with DNA. At least two populations of DAPI are represented, one bound to the DNA duplex, and one representing free DAPI.

MD simulations were used to investigate the interactions between alkyl imidazolium based ILs with. The results from MD simulations indicated that a fraction of IL cations always enter the DNA minor groove in a dilute solution of ILs. With increased IL concentration, the cationic headgroups lie near the DNA phosphates groups. Tail interactions are also observed where, at low IL concentration, the alkyl chains are arranged parallel to the DNA surface, and, with increased IL concentration, the alkyl chains are arranged perpendicular to the surface of DNA. Furthermore, with shorter alkyl chains, DNA–IL complexes form that show electrostatic attraction between the cation and DNA phosphates, π–π stacking interaction, and hydrophobic interaction between the alkyl chains and the DNA bases. The electrostatic, π–π stacking, and hydrophobic interactions are essential in the binding ILs to DNA.

## Data Availability

The data presented in this study are available on reasonable request from the corresponding author and are not publicly available.

## Supplementary Materials

The following supporting information can be downloaded, Figure S1: Structures of the imidazolium chloride ionic liquids, Figure S2: Steady-state emission spectra at various DAPI:DNA ratios, Figure S3: DAPI + [Im_16,1_]Cl in 10 mM tris buffer at pH = 7.0. Figure S4: DAPI + DNA + [Im_16,1_]Cl in 10 mM tris buffer at pH = 7.0., Figure S5: Excitation intensity ratios for DAPI and DAPI + DNA in 10 mM tris buffer at pH = 7.0 as a function of IL concentration, Figure S6: Center-of-mass (COM) radial distribution functions (RDF) for the [Im_4,1_]+ and [Im_10,1_]+ imidazolium rings around phosphate groups, minor, and major grooves of DNA duplex, Figure S7: Root-mean-square deviations from equilibration runs, Figure S8: Unsmoothed RDFs for tail-groove interactions in 20 mM [Im_16,1_]Cl, Table S1: Intensity decay fits for solutions of DAPI + [Im_16,1_]Cl, Table S2: Intensity decay fits for solutions of DAPI + DNA + [Im_16,1_]Cl.

## Sample Availability

Samples of the compounds are not available from the authors.
