# Supplementary material for: Interactions between a dsDNA Oligonucleotide and Imidazolium Chloride Ionic Liquids: Effect of Alkyl Chain Length, Part I
Source: Molecules. 2021 Dec 25;27(1):116. doi: 10.3390/molecules27010116 (PMC8746396; doi:10.3390/molecules27010116)
Supplement: Supplementary file 1 [file molecules-27-00116-s001.zip › molecules-1515815-supplementary.pdf]

Interactions between a dsDNA Oligonucleotide and Imidazolium Chloride Ionic Liquids: Effect of Alkyl

Chain Length, Part I.

Supplementary Materials

Fatemeh Fadaei [1,2], Michelle Seifert [3], Joshua Raymond [3], David Reha [1], Natallia Kulik [1], Babak Minofar [1,*] and Mark P. Heitz [3,*]

1 Laboratory of Structural Biology and Bioinformatics, Institute of Microbiology of the Czech Academy of Sciences, Zámek 136, 37333 Nove Hrady, Czech Republic; fadaei@nh.cas.cz (F.F.); reha@nh.cas.cz (D.Rˇ .); kulik@nh.cas.cz (N.K.)

2 Faculty of Science, University of South Bohemia in Cˇ eské Budeˇjovice, Branišovská 1645/31A, 37005 Ceske Budejovice, Czech Republic

3 Department of Chemistry and Biochemistry, SUNY Brockport, Brockport, NY 14420, USA; michelle.seifert@t-online.de (M.S.); jraym1@brockport.edu (J.R.R.)

* Correspondence: minofar@nh.cas.cz (B.M.); mheitz@brockport.edu (M.P.H.)

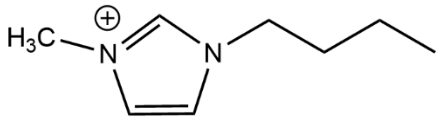

$Im_{4,1}^{+}$

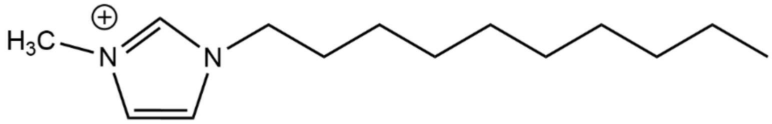

$Im_{10,1}^{+}$

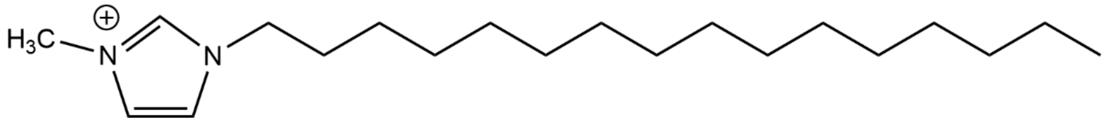

$Im_{16,1}^{+}$

Figure S1. Structures of 1-butyl-3-methylimidazolium chloride ([$Im_{4,1}$]Cl), 1-decyl-3-methylimidazolium chloride ([$Im_{10,1}$]Cl), and 1-hexadecyl-3-methylimidazolium chloride ([$Im_{16,1}$]Cl).

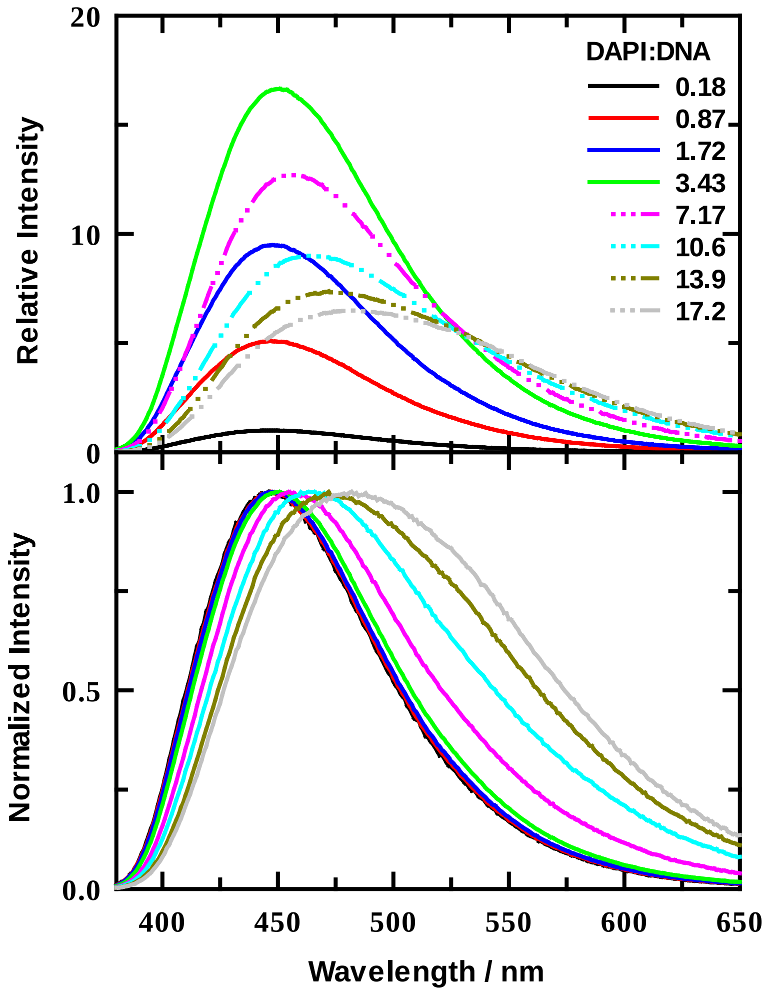

Figure S2. Steady-state emission spectra at various DAPI:DNA ratios. Arrows show this shift in the spectra as [DAPI] increases.

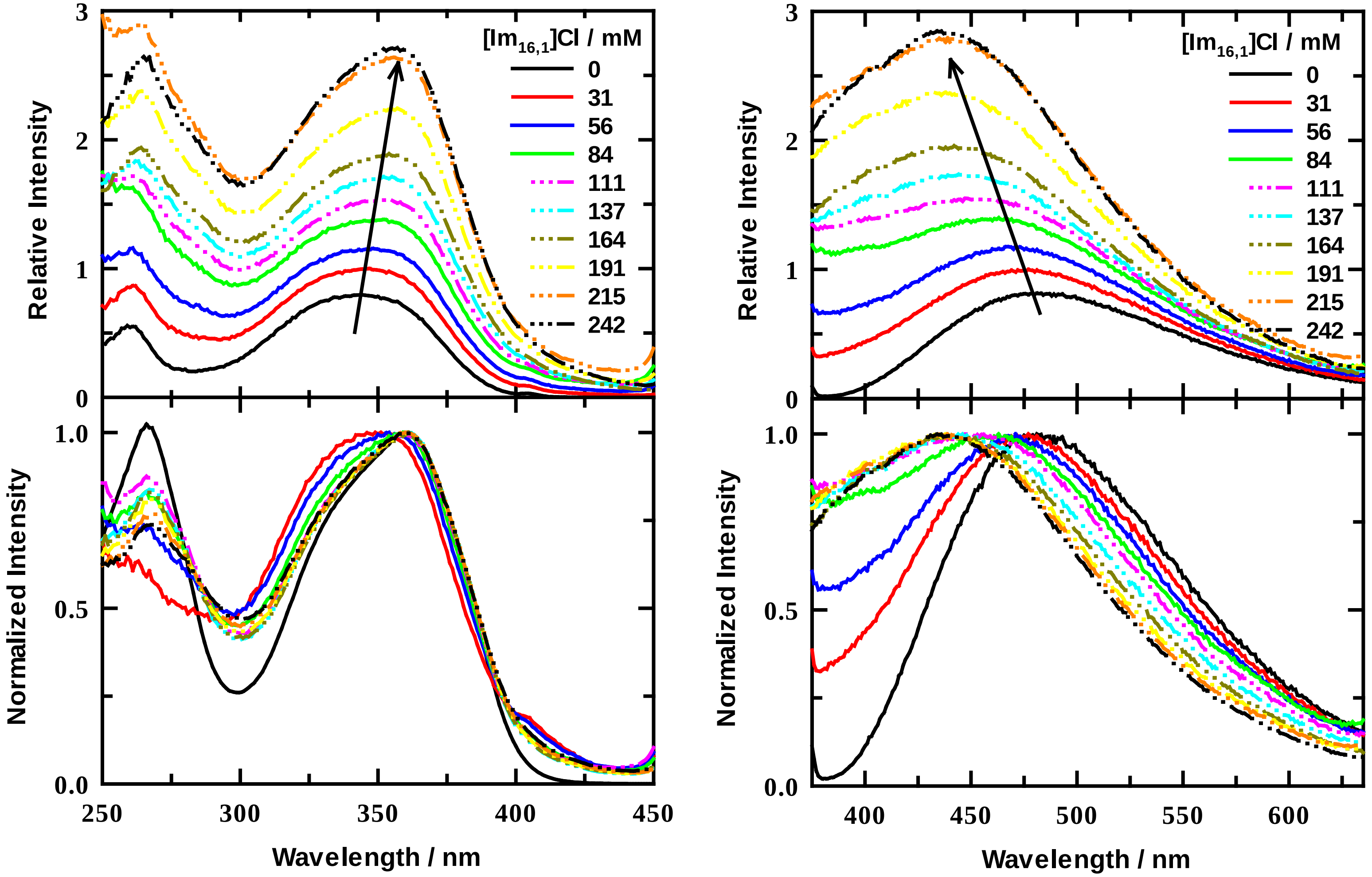

Figure S3. DAPI + [Im$_{16,1}$]Cl in 10 mM tris buffer at pH = 7.0. Upper Left Panel: excitation spectra normalized to the 31 mM spectrum. Lower Left Panel: individually normalized excitation. Right Panels: emission spectra. Arrows indicate shift direction.

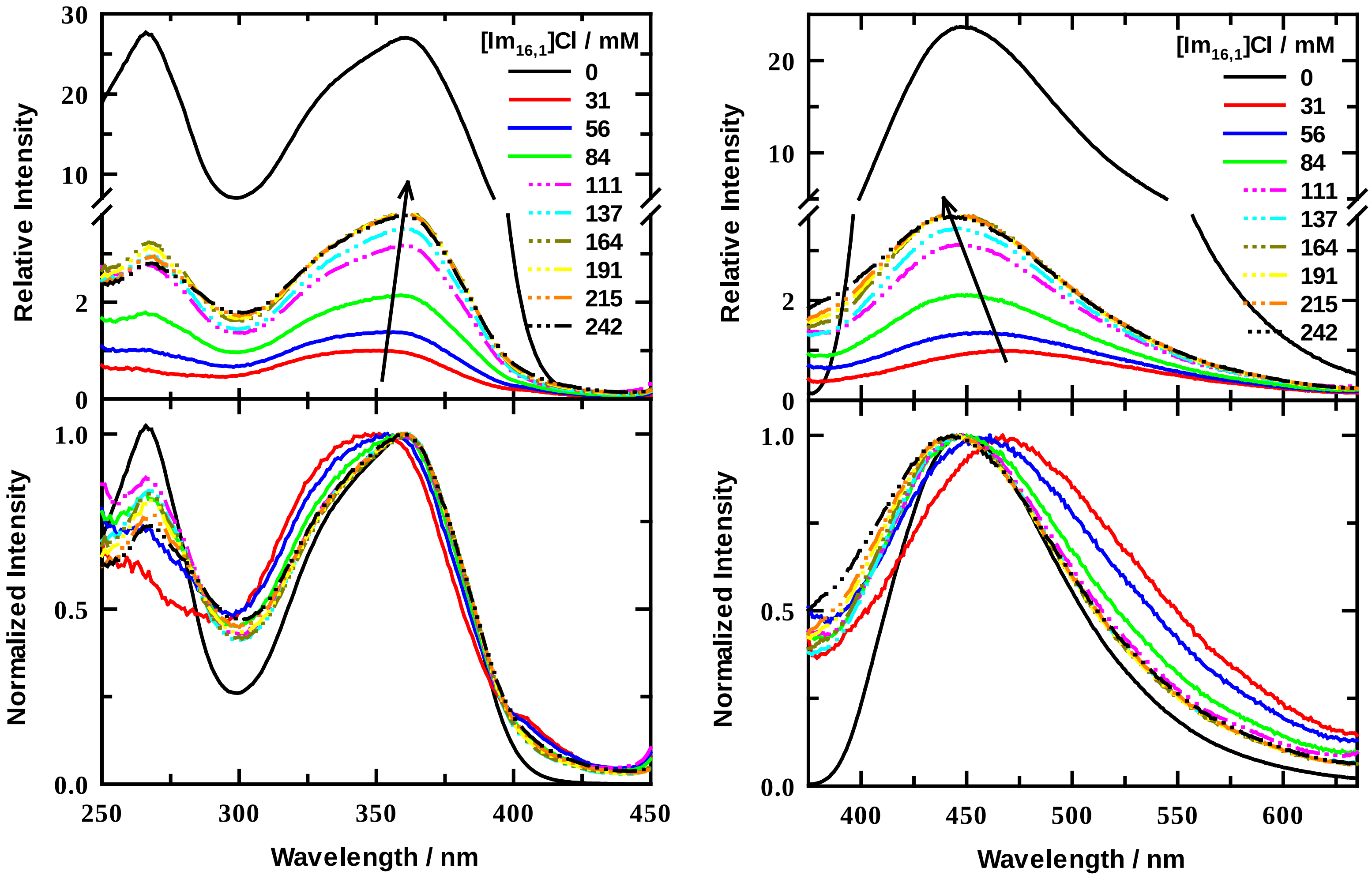

Figure S4. DAPI + DNA + [$Im_{16,1}$]Cl in 10 mM tris buffer at pH = 7.0. Left Upper Panel: excitation spectra normalized to the 31 mM spectrum. Left Lower Panel: normalized excitation. Right Panels: emission spectra. Arrows indicate shift direction.

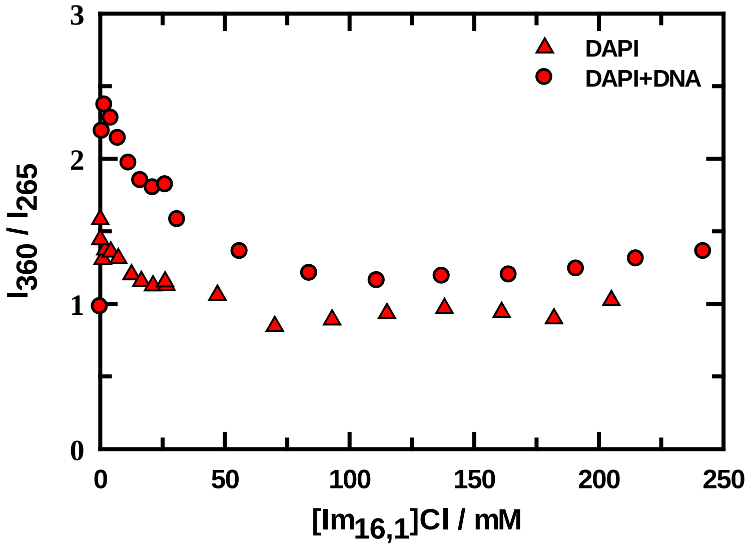

Figure S5. Excitation intensity ratios for DAPI and DAPI + DNA in 10 mM tris buffer at pH = 7.0 as a function of IL concentration.

Table S1. Results of the DAPI + $[Im_{16,1}]Cl$ Intensity Decay Fits.

$[Im_{16,1}]Cl$ / mM	f_1	f_2	τ_1 / ns	τ_2 / ns	χ_r^2	$\langle\tau\rangle$ / ns[a]
0	0.767 ± 0.005	0.232 ± 0.002	0.145 ± 0.001	2.55 ± 0.02	1.2	0.706 ± 0.008
0.1	0.777 ± 0.006	0.222 ± 0.002	0.145 ± 0.001	2.40 ± 0.02	1.2	0.647 ± 0.008
0.4	0.773 ± 0.006	0.226 ± 0.002	0.145 ± 0.001	2.36 ± 0.02	1.2	0.648 ± 0.008
0.9	0.769 ± 0.005	0.230 ± 0.002	0.147 ± 0.001	2.37 ± 0.02	1.1	0.661 ± 0.008
1.8	0.761 ± 0.005	0.238 ± 0.002	0.146 ± 0.001	2.41 ± 0.01	1.1	0.688 ± 0.008
3.2	0.750 ± 0.005	0.249 ± 0.002	0.147 ± 0.001	2.41 ± 0.02	1.2	0.712 ± 0.009
5.2	0.733 ± 0.005	0.266 ± 0.002	0.142 ± 0.001	2.39 ± 0.01	1.1	0.743 ± 0.008
7.1	0.721 ± 0.005	0.278 ± 0.002	0.143 ± 0.001	2.46 ± 0.01	1.2	0.790 ± 0.009
11	0.702 ± 0.005	0.297 ± 0.002	0.143 ± 0.001	2.49 ± 0.01	1.2	0.842 ± 0.009
28	0.634 ± 0.003	0.365 ± 0.002	0.155 ± 0.001	2.70 ± 0.01	1.2	1.154 ± 0.007

[a]The average lifetime is calculated using equation (2).

Table S2. Results of the DAPI + DNA + $[Im_{16,1}]Cl$ Intensity Decay Fits.

$[Im_{16,1}]Cl$ / mM	f_1	f_2	f_3	τ_1 / ns	τ_2 / ns	τ_3 / ns	χ_r^2	$\langle\tau\rangle$ / ns[a]
0	0.009 ± 0.001	0.049 ± 0.004	0.940 ± 0.005	0.25 ± 0.02	1.75 ± 0.13	3.79 ± 0.01	1.1	3.66 ± 0.02
0.01	0.013 ± 0.000	0.042 ± 0.002	0.943 ± 0.003	0.32 ± 0.02	1.73 ± 0.11	3.86 ± 0.01	1.0	3.72 ± 0.01
0.02	0.014 ± 0.001	0.053 ± 0.002	0.931 ± 0.003	0.30 ± 0.02	1.60 ± 0.08	3.84 ± 0.01	1.1	3.67 ± 0.01
0.05	0.022 ± 0.001	0.066 ± 0.003	0.910 ± 0.003	0.26 ± 0.01	1.45 ± 0.06	3.55 ± 0.01	1.1	3.34 ± 0.01
0.08	0.035 ± 0.001	0.056 ± 0.002	0.908 ± 0.003	0.19 ± 0.01	1.22 ± 0.05	3.51 ± 0.01	1.1	3.26 ± 0.01
0.1	0.045 ± 0.001	0.044 ± 0.002	0.910 ± 0.003	0.16 ± 0.01	1.10 ± 0.06	3.75 ± 0.01	1.0	3.47 ± 0.01
0.11	0.054 ± 0.001	0.067 ± 0.002	0.878 ± 0.003	0.17 ± 0.01	1.33 ± 0.05	3.47 ± 0.01	1.1	3.15 ± 0.01
0.21	0.099 ± 0.001	0.057 ± 0.002	0.842 ± 0.003	0.15 ± 0.01	1.16 ± 0.05	3.48 ± 0.01	1.1	3.02 ± 0.01
0.4	0.140 ± 0.002	0.025 ± 0.003	0.834 ± 0.004	0.14 ± 0.01	0.80 ± 0.10	3.68 ± 0.01	1.1	3.11 ± 0.01
0.9	0.199 ± 0.005	0.023 ± 0.004	0.776 ± 0.005	0.14 ± 0.01	0.63 ± 0.10	3.57 ± 0.01	1.1	2.81 ± 0.02
1.8	0.270 ± 0.006	0.027 ± 0.004	0.701 ± 0.006	0.14 ± 0.01	0.97 ± 0.13	3.53 ± 0.01	1.1	2.54 ± 0.02
3.5	0.360 ± 0.008	0.033 ± 0.005	0.606 ± 0.007	0.14 ± 0.01	1.28 ± 0.19	3.45 ± 0.02	1.1	2.19 ± 0.02
5.4	0.580 ± 0.012	0.043 ± 0.005	0.376 ± 0.005	0.14 ± 0.01	0.87 ± 0.10	3.30 ± 0.03	1.0	1.36 ± 0.02

[a]The average lifetime is calculated using equation (2).

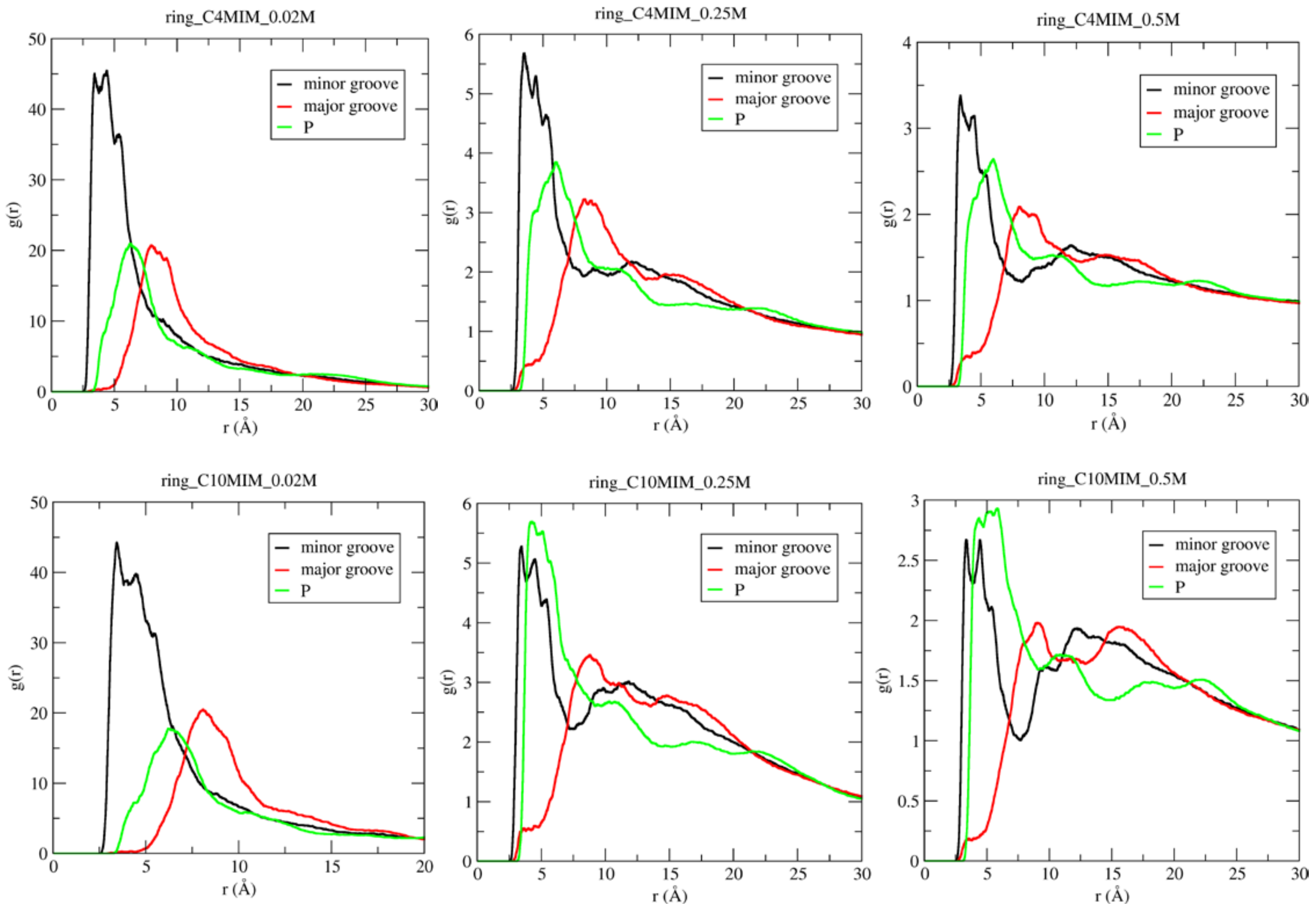

Figure S6. Center-of-mass (COM) radial distribution functions (RDF) for the $[Im_{4,1}]^+$ and $[Im_{10,1}]^+$imidazolium rings around phosphate groups, minor, and major grooves of DNA duplex in different IL concentration solutions. RDFs calculations are performed using the N7, O6, and O4 atoms for the electronegative sites in the major groove. Electronegative sites for the minor groove use N3 and O2 atoms. P atoms were used for the phosphate groups. IL concentrations are 20 mM (left panel), 250 mM (middle panel), and 500mM (right panel).

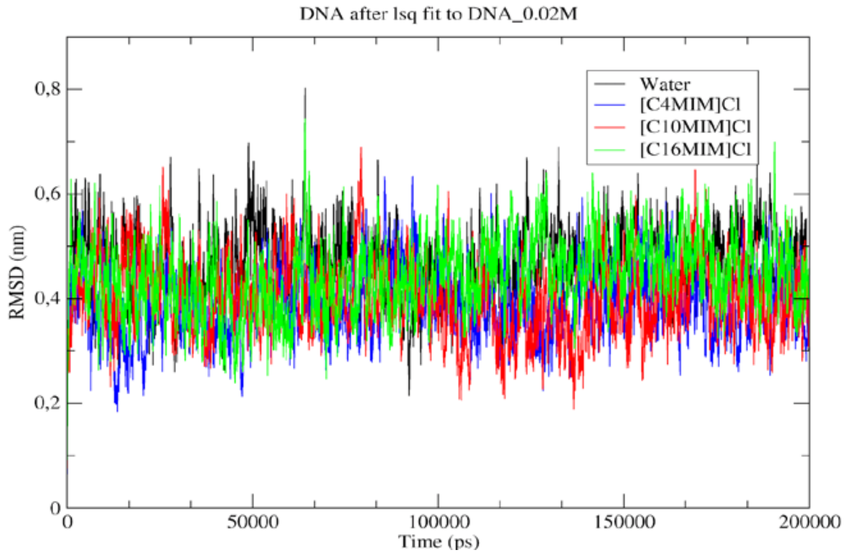

Figure S7. Computed root-mean-square deviation (RMSD) for DNA in water and 20 mM IL solutions, showing achievement of simulation equilibration.

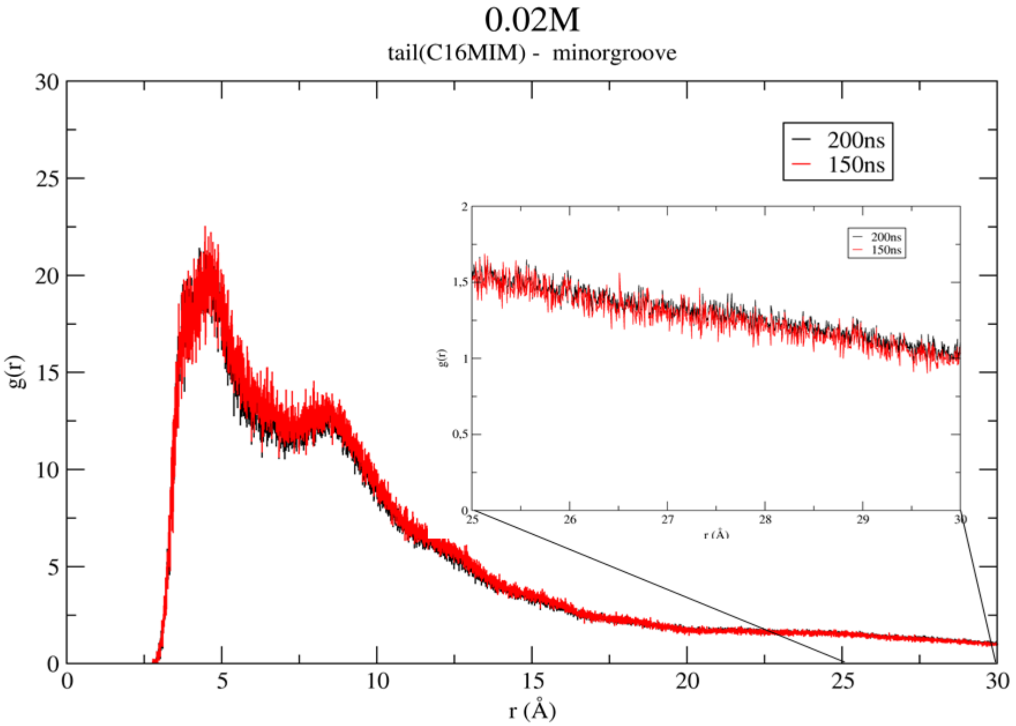

Figure S8. Unsmoothed radial distribution functions (RDF) for tail – groove interactions in 20 mM [$Im_{16,1}$]Cl, to show the actual fluctuations in the center-of-mass (COM) around the DNA minor grooves. The electronegative sites N3 and O2 atoms for the minor groove were used for these RDF calculations.

We note here that the bulk density is an average quantity, and as such it must be greater than the local density at some point in the system. However, we must mention that to fulfill such a criterion we would need to have solutions that show no aggregation. This is problematic here because for long alkyl chain imidazolium ILs, even in low concentration solutions (here 20 mM), aggregation appears in the simulations that influences the local density. Given that the aggregation concentration for $[Im_{16,1}]Cl$ is ~1 mM we are unable to accommodate this solution requirement.
